# Supplementary material for: What Makes a Rabbit Cute? Preference for Rabbit Faces Differs according to Skull Morphology and Demographic Factors
Source: Animals (Basel). 2019 Sep 26;9(10):728. doi: 10.3390/ani9100728 (PMC6826725; doi:10.3390/ani9100728)
Supplement: Supplementary file 1 [file animals-09-00728-s001.zip › Supplementary Material.docx]

Supplementary Materials for: What makes a rabbit cute? Preference for rabbit faces differ according to skull morphology and demographic factors

Naomi D. Harvey ^1,^ *, James Oxley ^2^, Giuliana Miguel-Pacheco ^1^, Emma M. Gosling ^3^ and Mark Farnworth ^4^

^1^ School of Veterinary Medicine and Science, The University of Nottingham, Sutton Bonington Campus, LE12 5RD, Leicestershire, UK

^2^ Independent Researcher, Measham, Swadlincote, Derbyshire DE12 7LQ, UK

^3^ APHA Scientific, Sutton Bonington, Loughborough, LE12 5RB Leicestershire, UK

^4^ School of Animal Rural & Environmental Sciences, Nottingham Trent University, Brackenhurst Campus, Southwell, NG25 0QF, Nottinghamshire, UK

***** Correspondence: naomi.harvey@nottingham.ac.uk

**Table S1.** Sources and copyright details for the 25 rabbit images used in this study.

| **Image ID** | **Source** | **Licence**  **Type** | **Photographer** |
| --- | --- | --- | --- |
| 1 | Authors own |  | Emma Gosling |
| 2 | <https://commons.wikimedia.org/wiki/File:Miniature_Lop_-_Side_View.jpg> | CC-BY-SA-3.0 | Miniaturelop |
| 3 | <https://commons.wikimedia.org/wiki/File:Cashmere_Lop.jpg> | CC-BY-SA-3.0 | Cashmerelop |
| 4 | <https://fr.wikipedia.org/wiki/Fichier:Lapin_b%C3%A9lier_J1.jpg> | CC-BY-SA-3.0 | Jamain |
| 5 | <https://commons.wikimedia.org/wiki/File:Netherland_Dwarf_On_Brick.jpg> | CC-BY-SA-3.0 | Erebus555 at en.wikipedia |
| 6 | <https://www.maxpixel.net/Netherland-Adorable-Dwarf-Rabbit-Cute-Bunny-2653648> | CC0 | N/A |
| 7 | <https://pixabay.com/en/hare-rabbit-black-grey-2175403/> | CC0 | N/A |
| 8 | <https://pxhere.com/en/photo/52479> | CC0 | N/A |
| 9 | Authors own |  | Emma Gosling |
| 10 | Authors own |  | Naomi Harvey |
| 11 | <https://pixabay.com/en/dwarf-rabbit-rabbit-nager-hare-pet-1124476/> | CC0 | N/A |
| 12 | <https://pixabay.com/en/hare-rabbit-dwarf-bunny-long-eared-581/> | CC0 | N/A |
| 13 | https://commons.wikimedia.org/wiki/File:OtisHavana.jpg | CC-BY-SA-3.0 |  |
| 14 | https://www.maxpixel.net/Rabbit-Pet-Dwarf-Rabbit-Dwarf-Bunny-Animal-World-2824961 | CC0 | N/A |
| 15 | Authors own |  | Emma Gosling |
| 16 | <https://pixabay.com/en/rabbit-bunny-animal-wildlife-wild-209609/> | CC0 | N/A |
| 17 | <https://pixabay.com/en/nature-rabbit-bunny-plush-white-73520/)> | CC0 | N/A |
| 18 | <https://pxhere.com/en/photo/643528> | CC0 | N/A |
| 19 | <https://pixabay.com/en/rabbit-dwarf-rabbit-hare-floppy-ear-55347/> | | |
| 20 | <https://pixabay.com/en/switzerland-rabbit-nature-2823537/> | CC0 | N/A |
| 21 | <https://pixabay.com/en/rabbit-white-ears-big-1733078/> | CC0 | N/A |
| 22 | <https://cs.wikipedia.org/wiki/Soubor:Teddykaninchen.jpg> | CC-BY-SA-4.0 | OxPhotographie |
| 23 | <https://pixabay.com/en/rabbit-bunny-u%C5%A1%C3%A1k-white-stunted-2288378/> | CC0 | N/A |
| 24 | <https://www.flickr.com/photos/134832191@N08/34849329843/in/photostream/> | CC BY 2.0 | Martyn Fletcher |
| 25 | <https://www.maxpixel.net/Rabbit-Cute-White-Background-Isolated-Bunny-Pet-740621> | CC0 | N/A |

**Univariate associations between preference ratings and rabbit characteristics**

The phenotypic characteristics assigned to the images (cephalic group, fur type, ear type and colour) all exhibited statistically significant associations with the preference score (Table S3). However, it must be remembered that the sample size in this study is considerable, so effect sizes and presence of visible differences in the score distribution should be favoured over p-value significance testing. Differences were seen in median preference ratings depending on ear type, with lop-eared rabbits scoring 1-unit lower on average than non-lop-eared rabbits, whilst no visible difference in the score distribution could be seen for fur type (long or short haired). Additionally, mixed colour and medium-light colour rabbits were preferred less compared to light, dark or grey rabbits, which scored an average of 1-unit higher in the preference ratings.

**Table S2.** Univariate results for associations between preference ratings (n=20858) for images of 25 different rabbit faces and phenotypic characteristics of the rabbit faces. *All images were presented in greyscale, so ‘colour’ indicates whether the rabbits fur appeared to be all uniform light, medium-light, grey, dark or whether it had a mixed colouring.

| **Variable** | **Type** | **Median** | **Interquartile Range** | **Test** | **Test statistic** | **P** |
| --- | --- | --- | --- | --- | --- | --- |
| Cephalic group | 1 Extreme-brachycephalism | 6 | 4 to 8 | Kruskal-Wallis | 26841 | <0.001 |
|  | 2 Moderate-brachycephalism | 7 | 5 to 8 |  |  |  |
|  | 3 Mild-brachycephalism | 7 | 6 to 9 |  |  |  |
|  | 4 Mesocephalic | 6 | 5 to 8 |  |  |  |
|  | 5 Mild-dolicocephalism | 7 | 5 to 8 |  |  |  |
|  | 6 Moderate-dolicocephalism | 5 | 3 to 7 |  |  |  |
| Ears | Lop-eared | 6 | 5 to 8 | Mann Whitney U | 3.31E+10 | <0.001 |
|  | Non-lop | 7 | 5 to 8 |  |  |  |
| Fur | Short-haired | 7 | 5 to 8 | Mann Whitney U | 3.22E+10 | <0.001 |
|  | Long-haired | 7 | 5 to 8 |  |  |  |
| Colouring* | Dark | 7 | 5 to 8 | Kruskal-Wallis | 2952 | <0.001 |
|  | Grey | 7 | 5 to 8 |  |  |  |
|  | Medium-light | 6 | 4 to 8 |  |  |  |
|  | Light | 7 | 5 to 8 |  |  |  |
|  | Mixed | 6 | 4 to 8 |  |  |  |

**Results from univariate analysis of demographic factors as predictors of preference for extremely brachycephalic rabbit images**

Considering only the responses to the sub-population of rabbit images rated as extremely-brachycephalic (six rabbits with a median cephalic rating of 1), respondents whose declared location was from a European country scored these rabbits as 1-unit lower on the preference scale than respondents from the rest of the world (Table S4). Increasing age was associated with a reduction in preference scores for this sub-category of rabbits (Fig. S1), as was working in a career related to animal health although the effect size was comparatively small. Whilst there was no difference in the median scores assigned by current rabbit owners and non-rabbit owners, current owners had a higher interquartile range (4 to 8 compared to 3 to 8 for non-owners). Education level was also associated with scores for this group, with people with advanced/postgraduate degrees typically scoring these rabbits 1-unit lower than all other groups.

**Table S3.** Univariate results for associations between preference ratings (n=20858) for images of 6 rabbit faces classified as being extremely-brachycephalic and phenotypic characteristics of the rabbit faces. Highlighted in bold are the categories that had a median score below the population-level median of 6 for this group.

| **Variable** | **Type** | **Median** | **Interquartile Range** | **Test** | **Test statistic** | **P** |
| --- | --- | --- | --- | --- | --- | --- |
| Continent | Not given | 6 | 4 to 8 | Kruskal-Wallis | 1125 | <0.001 |
|  | Africa | 6 | 4 to 8 |  |  |  |
|  | Asia | 6 | 4 to 8 |  |  |  |
|  | Oceania | 6 | 3 to 8 |  |  |  |
|  | **Europe** | **5** | **3 to 7** |  |  |  |
|  | North America | 6 | 4 to 8 |  |  |  |
|  | Latin America & Caribbean | 6 | 4 to 8 |  |  |  |
| Age | Not given | 6 | 4 to 8 | Kruskal-Wallis | 2189 | <0.001 |
|  | < 17 | 6 | 4 to 8 |  |  |  |
|  | 18 to 24 | 6 | 4 to 8 |  |  |  |
|  | 25 to 34 | 6 | 4 to 8 |  |  |  |
|  | **35 to 44** | **5** | **3 to 7** |  |  |  |
|  | **45 to 54** | **5** | **3 to 7** |  |  |  |
|  | **55 to 64** | **5** | **3 to 7** |  |  |  |
|  | **65 to 74** | **5** | **3 to 6** |  |  |  |
|  | **> 75** | **4** | **2 to 6** |  |  |  |
| Worked in an animal health related career | Not given | 6 | 4 to 8 | Kruskal-Wallis | 835 | <0.001 |
|  | Yes, in the past | 6 | 3 to 8 |  |  |  |
|  | No, never | 6 | 4 to 8 |  |  |  |
|  | **Yes** | **5** | **3 to 7** |  |  |  |
| Current rabbit owner | No | 6 | 3 to 8 | Kruskal-Wallis | 452 | <0.001 |
|  | Yes | 6 | 4 to 8 |  |  |  |
| Education level | Not given | 6 | 4 to 8 | Kruskal-Wallis | 1423 | <0.001 |
|  | **Advanced degree** | **5** | **3 to 7** |  |  |  |
|  | Bachelor’s degree | 6 | 3 to 8 |  |  |  |
|  | Associates degree | 6 | 4 to 8 |  |  |  |
|  | College courses, no degree | 6 | 4 to 8 |  |  |  |
|  | Trade/technical school | 6 | 3 to 8 |  |  |  |
|  | GSCE/graduated high school | 6 | 4 to 8 |  |  |  |
|  | No qualifications | 6 | 4 to 8 |  |  |  |


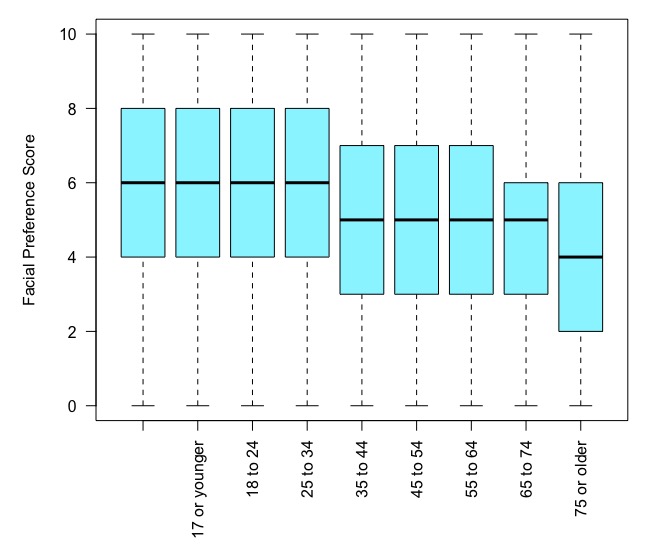


**Figure S1.** Boxplot showing the interquartile range and median preference ratings assigned to the 6 rabbit face images classified as extremely-brachycephalic by 20858 people separated by age group. The first boxplot is for those respondents who did not provide their age group.

**Table S4.** Preference rating summary statistics provided by 20,858 people for 25 rabbit face images sorted in descending order by mean rating. The rating scale ranged from 1 (“I do not like this rabbit at all”) to 10 (“this is my favourite type of rabbit”). See Figure A1 for images associated with each ID.

| **Image ID** | **Cephalic group** | **Median** | **Mean** | **SD** | **Minimum** | **Maximum** |
| --- | --- | --- | --- | --- | --- | --- |
| 13 | Mildly-BC | 8 | 8.1 | 1.8 | 0 | 10 |
| 19 | Mesocephalic | 7 | 7.3 | 1.9 | 0 | 10 |
| 14 | Mildly-BC | 7 | 7.1 | 2.0 | 0 | 10 |
| 16 | Mesocephalic | 7 | 7.0 | 1.9 | 0 | 10 |
| 11 | Moderately-BC | 7 | 6.9 | 2.2 | 0 | 10 |
| 24 | Mildly-DC | 7 | 6.9 | 1.9 | 0 | 10 |
| 2 | Extremely-BC | 7 | 6.8 | 2.6 | 0 | 10 |
| 20 | Mildly-DC | 7 | 6.8 | 2.1 | 0 | 10 |
| 12 | Mildly-BC | 7 | 6.7 | 2.4 | 0 | 10 |
| 15 | Mildly-BC | 7 | 6.7 | 1.9 | 0 | 10 |
| 7 | Moderately-BC | 7 | 6.6 | 2.4 | 0 | 10 |
| 9 | Mildly-BC | 7 | 6.6 | 2.2 | 0 | 10 |
| 3 | Moderately-BC | 7 | 6.5 | 2.4 | 0 | 10 |
| 22 | Mildly-BC | 7 | 6.5 | 2.5 | 0 | 10 |
| 10 | Mesocephalic | 6 | 6.3 | 2.1 | 0 | 10 |
| 25 | Mildly-DC | 6 | 6.3 | 2.3 | 0 | 10 |
| 1 | Extremely-BC | 6 | 5.7 | 2.4 | 0 | 10 |
| 17 | Mesocephalic | 6 | 5.7 | 2.2 | 0 | 10 |
| 6 | Extremely-BC | 6 | 5.5 | 2.7 | 0 | 10 |
| 5 | Extremely-BC | 5 | 5.4 | 2.7 | 0 | 10 |
| 8 | Extremely-BC | 6 | 5.4 | 2.8 | 0 | 10 |
| 18 | Moderately-DC | 5 | 5.3 | 2.4 | 0 | 10 |
| 4 | Extremely-BC | 5 | 5.2 | 2.9 | 0 | 10 |
| 21 | Moderately-DC | 5 | 4.9 | 2.8 | 0 | 10 |
| 23 | Mesocephalic | 5 | 4.9 | 2.5 | 0 | 10 |
